# Development and testing of culturally sensitive patient information material for Turkish, Polish, Russian and Italian migrants with depression or chronic low back pain (KULTINFO): study protocol for a double-blind randomized controlled trial

**DOI:** 10.1186/1745-6215-15-265

**Published:** 2014-07-04

**Authors:** Lars P Hölzel, Zivile Ries, Jördis M Zill, Levente Kriston, Jörg Dirmaier, Martin Härter, Isaac Bermejo

**Affiliations:** 1 Research Group on Psychotherapy and Health Services Research, Department of Psychiatry and Psychotherapy, Medical Center - University of Freiburg, Freiburg, Germany; 2 Department of Medical Psychology, University Medical Center Hamburg-Eppendorf, Hamburg, Germany; 3 Celenus Kliniken GmbH, Offenburg, Germany

**Keywords:** chronic disease, culture sensitive, depression, general practitioners, low back pain, migrants, patient information, randomized controlled trial

## Abstract

**Background:**

Many of the approximately 15 million people with a migration background living in Germany (19% of the population) are inadequately reached by existing healthcare provision. In the literature, the necessity for cultural adaptation of information material for patients with a migration background is often cited as a measure for improving healthcare.

In this study, culturally sensitive information material will be developed and evaluated for patients with a migration background and depression or chronic low back pain. In this respect, it will be examined whether culturally sensitive information material is judged as more useful by the patients than standard translated patient information without cultural adaptation.

**Methods/Design:**

The implementation and evaluation of culturally sensitive patient information material will occur in the framework of a double-blind randomized controlled parallel-group study in four study centres in Germany. Primary care patients with a Turkish, Polish, Russian or Italian migration background with a diagnosis of depressive disorder or chronic low back pain will be included and randomly allocated to the intervention group or the control group. In the intervention group, culturally sensitive patient information will be handed to the patient at the end of the physician consultation, while in the control group, standard translated patient information material will be provided. The patients will be surveyed by means of questionnaires following the consultation as well as after 8 weeks and 6 months. In addition to the primary outcome (subjective usefulness), several patient- and physician-rated secondary outcomes will be considered.

**Discussion:**

The study will provide an empirical answer to the question of whether persons with a migration background perceive culturally sensitive patient information material as more useful than translated information material without cultural adaptation.

**Trial registration:**

Deutsches Register Klinischer Studien (DRKS-ID) DRKS00004241 and Universal Trial Number (UTN) U1111-1135-8043.

## Background

A person’s healthcare is influenced by cultural and ethical values and attitudes as well as his or her migration experiences [1-3]. Language difficulties, a lack of information on the host country’s healthcare system and healthcare provision, deviating cultural beliefs, and a feeling of being misunderstood are central problems in the healthcare of persons with a migration background [4,5]. These barriers may be of elevated importance in chronic and multifactorially determined diseases, in which lifestyle changes are required for an enduring treatment success, for example, depression or chronic low back pain. To ensure successful changes in lifestyle, patients have to be adequately informed. An efficient way to inform patients is provided by written patient information material (for example, brochures), which increases knowledge about the illness and its treatment and is therefore a notable supplement to the consultation [6]. Patient information material can help the patient to develop a realistic perception of the course of the disease and increase his or her involvement [7]. It supports patients by informing them about sensible self-help activities and advising how to improve coping strategies [8]. Moreover, it helps patients to become aware of their own preferences when different treatment options are given and to make informed decisions.

When addressing patients with a migration background, merely translating patient information material into the patient’s mother tongue might not be sufficient to deal with cultural barriers. The understanding of the concepts of ‘health’ and ‘disease’ depends on the sociocultural context of a country, and therefore culturally sensitive information material that is designed to meet the unique needs of migrants has been called for [9]. Such material might help to improve the healthcare of persons with a migration background, by reducing cultural barriers and facilitating effective communication to help them to recognise and cope actively with diseases [9,10]. However, research on the effects of culturally sensitive patient information material is insufficient. To date, high-quality investigations focusing on the specific effect of culturally sensitive material compared with high-quality standard translations using a double-blind, randomized controlled multicentre design are lacking.

### Objectives

The aim of our study is to evaluate the effects of the provision of culturally sensitive information material to primary care patients with a migration background with depression or chronic low back pain in comparison with that of non-culturally adapted material. This will be assessed with regard to perceived usefulness of the material as judged by the patient (primary outcome) and other short- and medium-term cognitive, behavioural, clinical and healthcare-related outcomes.

## Methods/Design

The effects of a culturally sensitive version of written patient information material compared with a standard translated version will be evaluated in a double-blind, randomized controlled multicentre trial involving four study centres. Participating study centres are the Research Group on Psychotherapy and Health Services Research, Department of Psychiatry and Psychotherapy, Medical Center - University of Freiburg (primary study centre); the Department of Medical Psychology, University Medical Center Hamburg-Eppendorf; the German Foundation Against Depression (Stiftung Deutsche Depressionshilfe); and the Network of Physicians Oberhausen-Mülheim-Duisburg (Ärzte Netzverbund Oberhausen-Mülheim-Duisburg). The study was approved by the institutional review board of the University Medical Center Freiburg (number 375/10) and the local review boards responsible for local physicians (Ärztekammer Nordrhein, number 2012400; Sächsische Landesärztekammer, number EK-BR-74/12-1; Landesärztekammer Baden-Württemberg, number B-F-2012-065, and Ärztekammer Hamburg, number MC-307/12).

We chose depressive disorders and low back pain as medical indications as both conditions are among the leading causes of burden of disease worldwide [11]. Both conditions are often chronic, require an active involvement of patients in their own healthcare and are expected to be sensitive to cultural background. In addition, they are highly prevalent in German primary care [12,13].

To further increase generalizability, we chose to include patients with various migration backgrounds. Persons with a Turkish origin were included, as they constitute the largest group of migrants in Germany. Polish migrants are the group with the highest percentage of recent immigrants and the third largest group in total. Russian migrants are often late re-settlers with a German origin. Italian migrants are the largest group of European Union migrants and are among the traditional migrant workers in Germany.

The majority of patients are treated in primary care. Moreover, patient information plays a central role in primary care because it often serves as the source of first contact. General practitioners therefore have to provide initial information about illness and corresponding treatment options. Given the high numbers of patients treated in primary care and the central role of information material in this setting, we chose primary care as the setting for our study.

### Inclusion criteria

General practitioners will consecutively assess patients for eligibility. Adult primary care patients with a Turkish, Polish, Russian or Italian migration background and with unipolar depressive disorder or non-specific chronic low back pain will be included. Having a migration background is defined as being of non-German nationality, having a first language other than German, being born in another country, or having a parent who was born in a country other than Germany. To be included, patients have to report that they perceive their non-German origin as part of their identity. Unipolar depressive disorder is defined according to the International Classification of Disease, 10th revision (ICD-10: codes F32.xx, F33.xx, F34.1). In accordance with German national guidelines, chronic low back pain is defined as pain in the area beneath the costal arch, above the inferior gluteal folds, with or without referred leg pain, for more than 12 weeks and without signs of a specific cause (ICD-10 codes: M54.5, M54.8, M54.9). Potentially eligible patients will be identified using routine health records. Sociodemographic information will be confirmed within the consultation. Diagnosis will be based on clinical judgment.

### Exclusion criteria

To ensure a high external validity of our results, we have not formulated any exclusion criteria. However, owing to the type of intervention (written patient information material) and the response format (questionnaire), patients who are unable to read or respond to a questionnaire in the language of their respective migration background will not be eligible to participate in this study.

### Recruitment of general practitioners

General practitioners will be recruited by inviting academic training practices, practices of the cooperating networks and general practitioners (using public registers) to participate in the study. General practitioners will receive an allowance of €40.00 per patient recruited.

### Development of the interventions

In a first step, we will develop written patient information material for the indication of unipolar depression and chronic low back pain on the basis of current clinical practice guidelines (for depression, [12]; for chronic low back pain, [14]) in the German language. In addition to information on epidemiology, diagnostics and prognosis, treatment options and responsibilities of different healthcare providers will be explained using comprehensible language. The content of the developed patient information material will be discussed in an expert workshop with respect to accuracy and comprehensibility. To ensure the high quality of the material, we will include healthcare professionals (including at least one professional who was engaged in the development of the respective guideline) as well as patient representatives in the process. The final brochure comprises 12 pages and includes figures, pictures and illustrative case examples.

Second, the material will be translated by professional translators using the forward-backward translation procedure [15]. To this aim, the material will be translated by two independent translators. Based on the two versions, a third independent translator will develop a consensual version, and the consensual version will be translated back into German by a fourth independent translator. The original version and the back-translated version will be cross-checked by the third translator in cooperation with the research team. This process guarantees a high quality of the translated patient information material. The resulting material, termed the ‘standard translated version’, will be used as the control condition.

Third, cultural differences in the respective countries will be explored in order to develop the culturally sensitive version of the patient information material. The translated patient information material will be discussed with respect to its cultural appropriateness in four separate focus groups, one for each nationality. The focus groups will consist of four to ten people with a migration background. All focus groups will be conducted by the same moderators using a manual. The focus group sessions will be audio-recorded and transcribed. Two independent researchers will perform content analysis according to the methods of Mayring and Schreier [16,17]. Differences will be resolved by discussion with a third independent researcher. Based on these results, a culturally sensitive version of the patient information material will be developed. Cultural adaptations will primarily focus on presentation; the core information will remain unchanged.

Fourth, comprehensibility of the culturally sensitive version will be pilot-tested with two persons per migrant group. In this step, only definitely misleading or incomprehensible expressions will be modified. The resulting culturally sensitive version of the material will then be used in the intervention group.

### Administration

Patients will be recruited and informed of the study by the participating general practitioners within the consultation (-T_1_, enrolment, see Table 1). Informed consent will be sought from each participating patient. After informed consent has been provided, patients will receive a sealed envelope containing written patient information material in the form of a brochure and a questionnaire (T_0_, allocation). The contents of the envelopes (standard translated material versus culturally sensitive material) will be centrally randomized by means of a computer-based algorithm and stratified by physician, disease and migration background (Turkey, Poland, Russia, Italy) with varying block sizes, to ensure a balanced proportion of participants in the study conditions and to prevent predictability. Each physician, provided in advance with sets of envelopes, according to the disease and the migration background of the eligible patient, will simply hand the patient the next envelope from the appropriate group of envelopes. The envelopes will be prepared by the study centres, which will have no direct contact with patients. Blinding of general physicians and patients will be ensured through the use of sealed envelopes. Concomitant care will not be restricted in any way.

**Table 1:** Study schedule

	**Study period**				
	**Enrolment**	**Allocation**	**Post-allocation**		**Close-out**
**Time point**	**-T**_**1**_	**T**_**0**_	**T**_**1**_	**T**_**2**_	**T**_**3**_
**Enrolment**					
Eligibility screen	×				
Informed consent	×				
Allocation		×			
**Interventions**					
Culturally sensitive patient information material			×		
Standard translated patient information material			×		
**Assessments**					
**General practitioner rating**					
Diagnosis	×				
Treatment	×				
**Self-rating**					
Demographic information (age, sex, migration background, mother tongue, German language proficiency, education)*			×		
Acculturation (SMAS)			×		
Usefulness of information material (USE)			×	×	×
Knowledge of the disease*			×	×	×
Perceived cultural sensitivity*			×		
Behaviour change*				×	×
Illness perception (Brief-IPQ)			×	×	×
Symptom self-rating (PHQ-9, Core set)			×	×	×
Acceptance of the disease or treatment*			×	×	×
Adherence (MOS)				×	×
Satisfaction with physician (ZAPA)			×	×	×
Health care utilization*				×	×
Quality of life (WHO-5)			×	×	×

-T_1_, within the consultation; T_0_, after informed consent has been given; T_1_, after consultation; T_2_, 8 weeks; T_3_, 6 months; Brief-IPQ, Brief Illness Perception Questionnaire; MOS, Medical Outcomes Study (general adherence); PHQ-9, 9-item version of the Patient Health Questionnaire; SMAS, Stephenson Multigroup Acculturation Scale; USE, Usefulness Scale for Patient Information Material; WHO-5, WHO 5 Well-being Index; ZAPA, questionnaire assessing satisfaction with outpatient care, with a focus on patient participation; *, self-constructed items.

Patients in the intervention group will receive a culturally sensitive version and patients in the control group will receive a standard translated version of brochures. Patients will read the brochures after the end of the consultation, and will be asked to fill in the questionnaire and return it by post using a prepaid envelope (T_1_, after consultation). If patients do not respond, they will be reminded by phone (if possible) after 2 weeks and by post after 3 weeks. At 8 weeks (T_2_) and 6 months (T_3_) after the consultation, patients will be contacted by post and asked to fill in follow-up questionnaires. To ensure a sufficient response rate at each time point, patients will receive an unconditional reimbursement of €5.00, as this measure has been shown to be associated with an increased response rate [18].

### Primary outcome

The primary outcome will be the perceived usefulness of the written patient information material, as assessed by the patient using the Usefulness Scale for Patient Information Material (USE) following the consultation (T_1_). Patients will be blinded to their group assignment. This scale was developed in an independent inpatient population of 120 patients with depression or chronic low back pain. It is an instrument to measure the perceived usefulness of written patient information material from a patient’s point of view, and has nine items. The total score of the final version is a generic estimate, while the three subscales can be used to differentiate cognitive, emotional and behavioural aspects of usefulness. The USE has turned out to be a reliable and valid scale.

### Secondary outcomes

Perceived usefulness (USE) will additionally be assessed at T_2_ and T_3_, to investigate the perception of usefulness of the information material over time. Differences in the subscales will additionally be assessed. We will additionally assess the effects of the culturally sensitive material on knowledge of the disease (self-constructed items; at T_1_, T_2_, T_3_) and whether adapted patient information material is perceived as more culturally adequate (perceived cultural sensitivity; self-constructed items; T_1_). In addition, the level of implementation of behaviour promoted in the patient information material (behaviour change; self-constructed items; T_2_, T_3_), perception of disease (Brief Illness Perception Questionnaire, Brief-IPQ; T_1_, T_2_, T_3_[19]), symptoms of depression or back pain (Patient Health Questionnaire, PHQ-9, Core set; T_1_, T_2_, T_3_[20,21]), and acceptance of disease and the treatment (self-constructed items; T_1_, T_2_, T_3_) will be investigated. Finally, the effects of the culturally sensitive material on adherence (MOS general adherence items; T_2_, T_3_[22]), Satisfaction with the physician (ZAPA; T_1_, T_2_, T_3_[23]), healthcare utilization (self-constructed items; T_2_, T_3_) and quality of life (WHO 5 Well-being Index, WHO-5; T_1_, T_2_, T_3_[24]) will be assessed.

### Additional parameters

Data on the following demographic parameters will be collected via self-rating questionnaire: age, sex, migration background, mother tongue, German language proficiency and education. The acculturation of the patient will additionally be captured by means of a standardized instrument (Stephenson Multigroup Acculturation Scale, SMAS; T_1_[25]). Moreover, physicians will be asked about the patient’s diagnosis and treatment.

At the level of the general practitioner, we will assess general characteristics of the physician, such as age, sex, migration background, mother tongue and level of experience with other cultures via a self-rating questionnaire, to gain information on the generalizability of the results. At the level of the general practitioners’ practice, we will gather information on the number of practitioners in the practice, case load, consultations conducted in languages other than German, practice staff with a migration background, whether patient information material in languages other than German is already offered, and general practitioners’ estimated usefulness of patient information material for people with a migration background. Finally, we will assess the self-rated competence of the physician in dealing with persons with a migration background, the subjective need for education in dealing with persons with a migration background, the perceived impact of interaction problems and other barriers to treating persons with a migration background.

### Statistical analyses

The primary outcome (usefulness of written patient information material; USE) will be analyzed by a blinded statistician with a one-sided *t* test comparing the scores on the USE scale between the intervention and the control group. Secondary interval-scaled outcomes will be tested using *t* tests and analysis of covariance (ANCOVA, if baseline measurement of the outcome is available). In additional analyses, we will include acculturation (SMAS) as covariate and conduct subgroup analyses regarding disease, migration background, symptom severity and acculturation. The primary analysis will be performed according to the intention-to-treat principle, including all randomized participants. Missing data will be handled by applying linear mixed models and expectation-maximisation imputation. Secondary analyses will be performed in available cases.

### Sample size

To be able to identify a small intervention effect (Cohen’s *d* of 0.3, which corresponds to a difference of 6 points in the USE (assuming a standard deviation of 20)) with a Type I error of 0.05 and a power of 80%, a total sample size of 280 patients (140 per group) is needed for statistical analysis. Taking into account a dropout rate of about 40% (patients who give informed consent but do not return the questionnaire by post), 480 patients shall be recruited. In the intention-to-treat analysis, the larger number of patients is expected to be compensated by a dilution of effects, meaning that the power will be approximately the same as in the available case analysis.

## Discussion

Our study will provide information on the effects of culturally sensitive patient information material in primary care on central patient-reported outcomes, such as perceived usefulness, disease-related knowledge, behaviour change, perception of disease, illness symptoms, acceptance of disease and treatment, adherence, satisfaction with the physician, health service utilization, and quality of life*.* This will be the first study to use a double-blind, randomized controlled multicentre design to evaluate the effects of culturally sensitive information material. By blinding both patients and the responsible physician, we are likely to control bias caused by expectations. The inclusion of different diseases and migration groups and the recruitment in multiple centres in different areas in Germany should ensure a high external validity of the results.

A central challenge of our study is the complexity of the intervention. The term ‘cultural adaptation’ is not consistently defined in the literature and there are many different ways in which patient information material could be adapted. Therefore, results might be specific to the type of cultural adaptation chosen for our study. However, we aim to describe the process of our cultural adaptation in detail and make it publicly available, to enable a replication and critical discussion of our results. We will take several measures to assure a high quality of the cultural adaptation process and the resulting culturally sensitive patient information material. To ensure a high external validity, only people with a migration background who are living in Germany will be invited to participate in the focus groups. All focus group sessions will be conducted by the same moderators using manuals to ensure high comparability. A high objectivity of our results will be ensured by the use of audio-recording and transcription of the focus group sessions and by the involvement of at least two independent researchers in the analyses of the results.

Potential limitations of the study are that the inclusion criteria (especially the diagnosis) are exclusively based on the clinical judgment of the general practitioners. For feasibility reasons, we chose to include patients regardless of the reason for consultation and we did not specify the timing in the disease course when the patients are eligible. This may dilute the effects of the intervention. Another limitation is that patients have to self-administer a high number of scales. This might hinder the feasibility of the trial and reduce the response rate. Moreover, the use of several self-constructed scales may be critical, as validity and reliability are unknown for some of the scales.

It has often been stressed that cultural adaptation of patient information material is crucial for improving the healthcare of patients with a migration background. However, high-quality studies dealing with this issue are largely lacking. Our study might therefore close an important knowledge gap. The results of our study may have a major impact on health services for patients with a migration background, as they will enable the incremental effect of culturally sensitive patient information material compared with high-quality standard translated material in primary care to be estimated.

## Trial status

The first patient was enrolled in June 2013. At the time of manuscript submission, participants were still being recruited.

## Abbreviations

ANCOVA: Analysis of covariance; Brief-IPQ: Brief Illness Perception Questionnaire; ICD-10: International Classification of Disease, 10th revision; MOS: Medical Outcomes Study; PHQ-9: 9-item version of the Patient Health Questionnaire; SMAS: Stephenson Multigroup Acculturation Scale; USE: Usefulness Scale for Patient Information Material; WHO: World Health Organization; WHO-5: WHO 5 Well-being Index; ZAPA: Questionnaire assessing satisfaction with outpatient care, with focus on patient participation.

## Competing interests

The authors declare that they have no competing interests.

## Authors’ contributions

All authors made substantial contributions to the conception and design. LK, LPH and IB planned the statistical analyses. LPH drafted the first version of the manuscript. ZR, JMZ, LK, JD, IB and MH revised the manuscript for important intellectual content. All authors read and approved the final manuscript.
